# Coupling Rare-Earth Complexes with Carbon Dots via Surface Imprinting: A New Strategy for Spectroscopic Cu^2+^ Sensors

**DOI:** 10.3390/molecules30193967

**Published:** 2025-10-02

**Authors:** Zuoyi Liu, Bo Hu, Minjia Meng

**Affiliations:** 1 School of Chemistry and Chemical Engineering, Jiangsu University, Zhenjiang 212013, China; 2 South China Institute of Environmental Sciences, Ministry of Ecology and Environment, Guangzhou 510000, China

**Keywords:** ion-imprinted polymer, ratiometric fluorescence, Cu^2+^ detection, visual sensor

## Abstract

A surface molecularly imprinted ratiometric fluorescent sensor (Eu/CDs@SiO_2_@IIPs) was constructed for the selective and visual detection of Cu^2+^. The sensor integrates blue-emitting carbon dots as an internal reference and a custom-designed Eu(III) complex, Eu(MAA)_2_(2,9-phen), as both the functional and fluorescent monomer within a surface-imprinted polymer layer, enabling efficient ratiometric fluorescence response. This structural design ensured that all fluorescent monomers were located at the recognition sites, thereby reducing background fluorescence interference and enhancing the accuracy of signal changes. Under optimized conditions, the sensor exhibited a detection limit of 2.79 nM, a wide linear range of 10–100 nM, and a rapid response time of 3.0 min. Moreover, the uncoordinated nitrogen atoms in the phenanthroline ligand improved resistance to interference from competing ions, significantly enhancing selectivity. Practical applicability was validated by spiked recovery tests in deionized and river water, with results showing good agreement with ICP-MS analysis. These findings highlight the potential of Eu/CDs@SiO_2_@IIPs as a sensitive, selective, and portable sensing platform for on-site monitoring of Cu^2+^ in complex water environments.

## 1. Introduction

Copper, as one of the earliest collected and utilized metals, has been widely applied in industry and agriculture. However, with the mining of copper ores and the discharge of copper-containing wastewater, copper ions continuously accumulate in aquatic environments and, due to their poor biodegradability, gradually bioaccumulate through the food chain and eventually enter the human body [1]. Divalent copper ions (Cu^2+^) are the predominant form of copper in aquatic systems, and their excessive accumulation can cause protein denaturation, leading to liver and kidney dysfunction, Alzheimer’s disease, and various cancers [2,3]. In recent years, with the aggravation of copper pollution, environmental monitoring and discharge standards for Cu^2+^ have become increasingly stringent, placing higher demands on current detection methods [4]. Traditional methods, such as inductively coupled plasma mass spectrometry [5], atomic absorption spectroscopy [6], spectrophotometry [7,8], and electrochemical techniques [9], although offering high sensitivity and selectivity, rely on expensive instrumentation, complicated procedures, and laborious sample pretreatment, which limit their ability to meet the increasing demand for frequent detection.

Among various emerging detection techniques, fluorescence analysis has gradually become an ideal choice for the rapid detection of Cu^2+^ owing to its high sensitivity, fast response, and visualization capability [10,11,12,13,14]. In particular, ratiometric fluorescence sensors, which rely on the self-calibration ability of dual- or multi-emission signals, can not only enhance color change effects but also effectively eliminate interference caused by fluctuations in light source intensity and probe concentration. Therefore, they are considered powerful tools for achieving highly sensitive visual detection [15,16,17,18,19]. However, due to the competition from various metal ions in aquatic environments, conventional fluorescent probes still suffer from poor selectivity, which requires molecular-level structural design to improve their recognition ability [20,21,22,23,24].

In recent years, rare-earth elements have attracted wide attention in fluorescence detection due to their unique electronic structures and optical properties. Trivalent europium ions (Eu^3+^), with their narrow red emission bands, long lifetimes, and large Stokes shifts, offer great potential for constructing high-performance fluorescent probes [25,26]. However, rare-earth complexes alone often exhibit low quantum efficiency, poor stability, and limited selectivity, which restrict their direct application in Cu^2+^ detection under complex environmental conditions [27]. Meanwhile, carbon dots (CDs), as a new class of fluorescent nanomaterials, possess excellent photostability and water solubility, and are frequently used as internal references, providing an ideal platform for constructing ratiometric fluorescence systems [28,29,30,31,32,33,34]. Therefore, functional integration of Eu^3+^ complexes with CDs and their precise localization at imprinted recognition sites are key to enhancing detection performance.

In the design of ratiometric fluorescence sensors, the customization of fluorescent functional monomers is a key strategy to overcome the limitations of weak response signals and the spatial separation between recognition and emission sites in traditional systems [35,36,37,38,39]. In conventional doped materials, fluorescent molecules are often randomly distributed, resulting in a limited number of effective response sites and susceptibility to interference from competing ions [40]. To address this issue, a lanthanide-based fluorescent functional monomer with both sensing and ion-recognition abilities was constructed and introduced into a pre-polymerization system via surface imprinting technology. In this way, the fluorescent centers were spatially coupled with the specific recognition sites, enabling direct fluorescence responses of Eu^3+^ complexes during Cu^2+^ binding, thereby significantly enhancing the ratiometric fluorescence change and improving selectivity.

**Figure 1:**
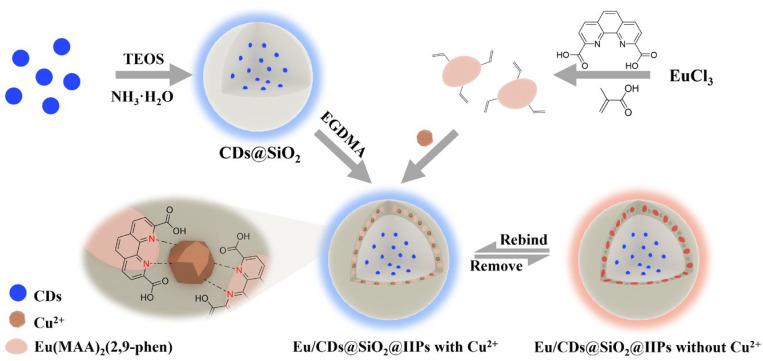
Preparation process diagram of Eu/CDs@SiO_2_@IIPs.

Based on this concept, a Eu/CDs@SiO_2_@IIPs ratiometric fluorescence sensor was constructed, in which the blue emission of CDs served as the internal reference and the red emission of Eu^3+^ complexes acted as the responsive signal, forming a dual-emission ratiometric fluorescence system (as shown in Figure 1). Systematic studies on its recognition ability and anti-interference performance toward Cu^2+^ confirmed the advantages of customized fluorescent functional monomers in improving selectivity and visualization. This work provides a new approach for integrating rare-earth functional monomers with ion-imprinting technology and offers a feasible solution for on-site detection of Cu^2+^ in complex aqueous environments.

## 2. Results and Discussion

### 2.1. Characterization of Eu(MAA)_2_(2,9-phen)

**Figure 2:**
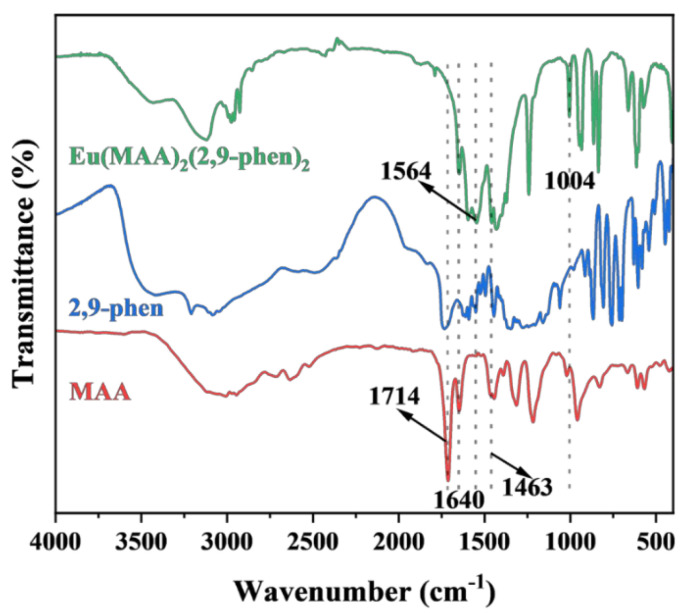
FT-IR spectra of MAA, 2,9-phenand Eu(MAA)_2_(2,9-phen).

To investigate the coordination mode and stoichiometry between Eu^3+^ ions and the ligands in the rare-earth complex, FT-IR spectroscopy and elemental analysis were employed. Figure 2 shows the infrared spectra of MAA, 2,9-phen, and the Eu complex. For the ligands MAA and 2,9-phen, the C=O stretching vibration at 1714 cm^−1^ and the asymmetric C–O stretching vibration at 1254 cm^−1^ are attributed to the carboxyl groups. In the Eu complex, the disappearance of the C=O stretching band at 1714 cm^−1^ indicates that the carboxyl groups are coordinated with Eu^3+^. The characteristic peak of the C=C double bond at 1640 cm^−1^ observed in both MAA and the Eu complex demonstrates that the C=C bonds of MAA did not participate in coordination, suggesting that the Eu complex can act as a fluorescent functional monomer for imprinting polymerization. Furthermore, the –COO^−^ stretching vibrations at 1564 cm^−1^ and 1463 cm^−1^ confirm the involvement of –COOH groups in coordination. In contrast, the N=N stretching band at 1004 cm^−1^ in the Eu complex indicates that the nitrogen atoms on the heteroaromatic ring of 2,9-phen did not participate in coordination.

From the FT-IR data, it can be concluded that the ligand 2,9-phen participates in coordination, as indicated by the disappearance of its characteristic carboxyl peaks, suggesting that both carboxyl groups are fully involved in coordination. The presence of the C=C band in the Eu complex further confirms that MAA also participates in the coordination process. Considering the common octacoordination of Eu^3+^, the stoichiometric ratio of Eu^3+^, MAA, and 2,9-phen in the complex was assumed to be 1:2:1. Elemental analysis was then performed to determine the composition of the Eu complex. As shown in Table 1, the experimental results of organic elemental analysis were consistent with the calculated values, confirming that the coordination structure of the complex can be assigned as Eu(MAA)_2_(2,9-phen).

### 2.2. Structural and Physicochemical Characterization of Eu/CDs@SiO_2_@IIPs

**Figure 3:**
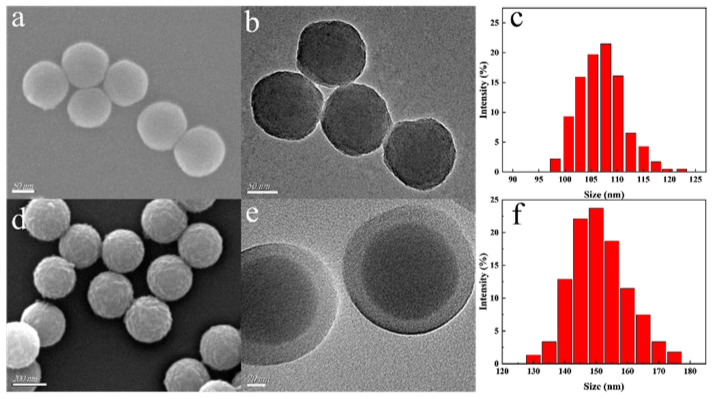
SEM images of CDs@SiO_2_ (**a**) and Eu/CDs@SiO_2_@IIPs (**d**); TEM images of CDs@SiO_2_ (**b**) and Eu/CDs@SiO_2_@IIPs (**e**); Dynamic light scattering size distribution of CDs@SiO_2_ (**c**) and Eu/CDs@SiO_2_@IIPs (**f**).

Figure 3 presents the SEM images, TEM images, and particle size analysis of the products obtained during the preparation of Eu/CDs@SiO_2_@IIPs. As shown in Figure 3a,c, SEM and TEM images of CDs@SiO_2_ display uniform size distribution, smooth surfaces, and an average diameter of about 100 nm. The TEM image further reveals quantum dot-sized CDs embedded inside the SiO_2_ matrix, indicating that the reverse microemulsion method successfully encapsulated CDs within the SiO_2_ nanospheres. Figure 3b,d shows the SEM and TEM images of Eu/CDs@SiO_2_@IIPs prepared by the surface imprinting method. As observed in Figure 3b, Eu/CDs@SiO_2_@IIPs exhibit good uniformity and monodispersity, with rougher surfaces compared to CDs@SiO_2_ and an increased particle diameter of approximately 150 nm. In addition, the TEM image in Figure 3d clearly shows a distinct core–shell structure, with an imprinting layer thickness of about 20 nm. These results confirm that Eu/CDs@SiO_2_@IIPs possess a core–shell architecture and that the imprinting layer was successfully polymerized on the surface of CDs@SiO_2_.

**Figure 4:**
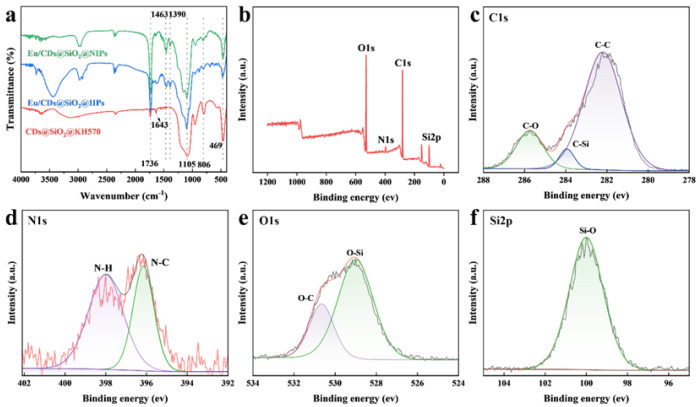
FT-IR spectra (**a**) of CDs@SiO_2_@KH570 (red), Eu/CDs@SiO_2_@IIPs (blue) and Eu/CDs@SiO_2_@NIPs (green); XPS spectra of Eu/CDs@SiO_2_@IIPs: full range (**b**), C1s (**c**), N1s (**d**), O1s (**e**) and Si2p (**f**).

To investigate the chemical composition of Eu/CDs@SiO_2_@IIPs, FT-IR spectroscopy was employed to characterize the products obtained during the reaction process. Figure 4a shows the infrared spectra of CDs@SiO_2_@KH570, Eu/CDs@SiO_2_@IIPs, and Eu/CDs@SiO_2_@NIPs. As observed, all samples exhibited three characteristic peaks at 1105 cm^−1^, 806 cm^−1^, and 469 cm^−1^, corresponding to the deformation and stretching vibrations of Si–O–Si bonds. In CDs@SiO_2_@KH570, the characteristic C=C peak at 1643 cm^−1^ and the C=O peak at 1736 cm^−1^ confirm the successful modification of KH570 on the surface of CDs@SiO_2_. In both IIPs and NIPs, the –COO^−^ stretching vibrations at 1463 and 1390 cm^−1^ are attributed to the coordinated carboxyl groups from the fluorescent functional monomer Eu(MAA)_2_(2,9-phen). These results demonstrate that CDs were successfully encapsulated within SiO_2_ particles and that both imprinted and non-imprinted polymers were successfully grafted onto the surface of the silica-based substrate.

Figure 4b shows the XPS survey spectrum of Eu/CDs@SiO_2_@IIPs. The photoelectron peaks at 100.08 eV, 281.82 eV, 397.13 eV, and 529.29 eV correspond to Si2p, C1s, N1s, and O1s, respectively, confirming that Eu/CDs@SiO_2_@IIPs are composed of these four elements. As shown in Figure 4c, the high-resolution C1s spectrum displays three peaks at 282.2 eV, 283.95 eV, and 285.75 eV, which can be assigned to C–C, C–Si, and C–O bonds. In Figure 4d, the peaks at 396.15 eV and 398.00 eV correspond to N–C and N–H bonds, which can be attributed to the heteroaromatic ring of the fluorescent functional monomer Eu(MAA)_2_(2,9-phen). In Figure 4e, the peaks at 529.00 eV and 530.65 eV are assigned to O–Si and O–C bonds, while the strong Si–O peak at 100.0 eV in Figure 4f originates from the silica framework. Taken together, the XPS results are consistent with the FT-IR data, confirming the successful synthesis of Eu/CDs@SiO_2_@IIPs.

### 2.3. Optimisation of Fluorescence Detection Conditions

**Figure 5:**
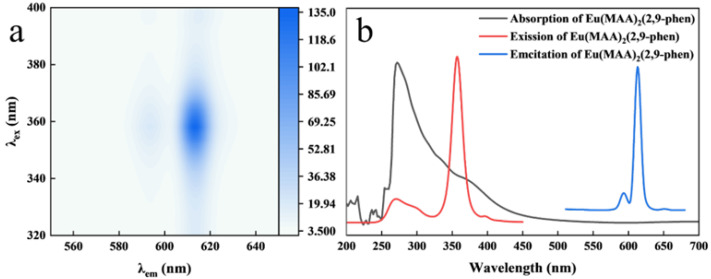
Fluorescence contour map (**a**) and combination diagram (**b**) of absorption spectrum, fluorescence excitation spectrum, and fluorescence emission spectrum of Eu(MAA)_2_(2,9−phen).

In the ratiometric fluorescence system of Eu/CDs@SiO_2_@IIPs, Eu(MAA)_2_(2,9-phen) and CDs serve as the responsive and internal reference signals, respectively; thus, the choice of an appropriate excitation wavelength is critical for the detection system. Figure 5a shows the fluorescence contour map of Eu(MAA)_2_(2,9-phen) under different excitation wavelengths. The emission peak at 614 nm remained unchanged regardless of the excitation wavelength. As shown in Figure 5b, the UV absorption, excitation, and emission spectra of Eu(MAA)_2_(2,9-phen) reveal a UV absorption peak at 272 nm. Based on the emission data, the optimal excitation wavelength of Eu(MAA)_2_(2,9-phen) was determined to be 357 nm. The optimal excitation wavelength of CDs has been discussed in Section 2.3, where it was found to be 297 nm. Although 357 nm is not the best excitation wavelength for CDs, they still emit detectable blue fluorescence under this excitation. Moreover, 357 nm is close to the commercial UV lamp wavelength of 365 nm, making it more practical for studying the spectral data of the ratiometric fluorescence system and understanding the visual response of Eu/CDs@SiO_2_@IIPs toward Cu^2+^. Considering that Eu(MAA)_2_(2,9-phen) exhibits maximum excitation at 357 nm, this wavelength was chosen for the excitation of Eu/CDs@SiO_2_@IIPs.

**Figure 6:**
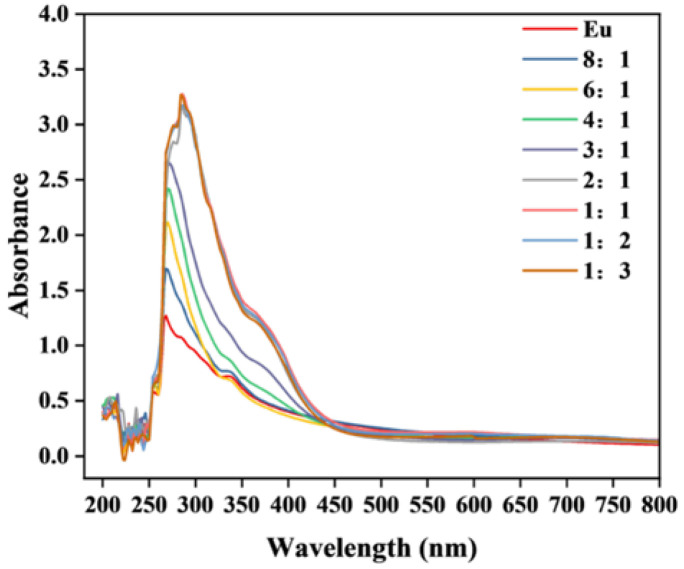
UV absorption spectra of Eu(MAA)_2_(2,9−phen) and Eu(MAA)_2_(2,9−phen) with Cu^2+^ at different molar ratios.

The optimal dosage of the fluorescent functional monomer should take into account the interaction between the template ions and the functional monomers. To investigate the optimal ratio of functional monomer to template ion in the Eu/CDs@SiO_2_@IIPs system, UV absorption spectra were recorded for Eu(MAA)_2_(2,9-phen) alone and for Eu(MAA)_2_(2,9-phen) mixed with Cu^2+^ at molar ratios of 8:1, 6:1, 4:1, 3:1, 2:1, 1:1, 1:2, and 1:3. In these measurements, the initial concentrations of Eu(MAA)_2_(2,9-phen) and Cu^2+^ at the 1:1 molar ratio were both 0.02 mol L^−1^. As shown in Figure 6, the absorption band at 272 nm for Eu(MAA)_2_(2,9-phen) corresponds to the B band, arising from the π–π* transition of the heteroaromatic ring in 2,9-phen. Upon the addition of Cu^2+^, this absorption band exhibited a red shift, which ceased to change significantly once the molar ratio of Eu(MAA)_2_(2,9-phen) to Cu^2+^ reached 2:1. This effect can be attributed to the coordination interaction between Cu^2+^ and the nitrogen atoms in the heteroaromatic ring of 2,9-phen. Therefore, the optimal ratio of functional monomer to template ion in the Eu/CDs@SiO_2_@IIPs system was determined to be 2:1.

**Figure 7:**
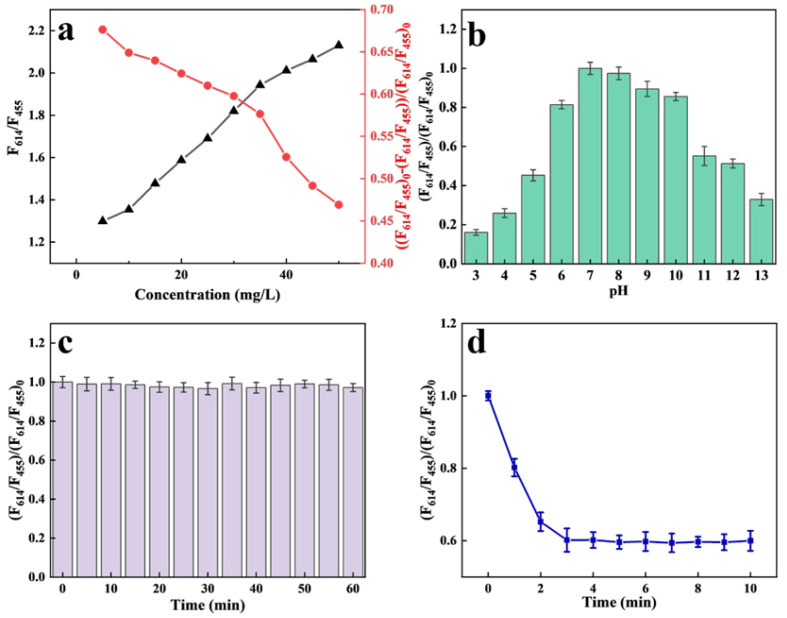
(**a**) Concentration−dependent fluorescence response of Eu/CDs@SiO_2_@IIPs; (**b**) Influence of pH on fluorescence intensity; (**c**) Fluorescence stability within 60 min; (**d**) Time−dependent fluorescence quenching in the presence of 50 nM Cu^2+^.

Eu/CDs@SiO_2_@IIPs were dispersed in aqueous media at concentrations of 5–50 mg/L in order to identify the optimal working concentration for detection. Fluorescence spectra were recorded using a spectrofluorometer, measuring both the initial fluorescence intensity ratio (F_614_/F_455_)_0_ and the ratio after the addition of 50 nM Cu^2+^ (F_614_/F_455_). The optimal concentration was evaluated based on the relative quenching efficiency, expressed as ((F_614_/F_455_)_0_ − (F_614_/F_455_))/(F_614_/F_455_)_0_. As shown in Figure 7a, (F_614_/F_455_)_0_ increased with the initial concentration of Eu/CDs@SiO_2_@IIPs, and no decrease in fluorescence intensity was observed even at 50 mg/L. The quenching efficiency ((F_614_/F_455_)_0_ − (F_614_/F_455_))/(F_614_/F_455_)_0_ reflects the extent of fluorescence quenching caused by Cu^2+^. For optimal detection performance, higher quenching efficiency values are desirable. However, as the initial concentration of Eu/CDs@SiO_2_@IIPs increased, the quenching efficiency gradually decreased and reached a critical point in the range of 30–35 mg/L. By weighing sensitivity against overall performance, 31 mg/L was identified as the most appropriate working concentration of Eu/CDs@SiO_2_@IIPs in the detection solution.

An investigation was conducted into how pH affects the fluorescence intensity of Eu/CDs@SiO_2_@IIPs. A series of 31 mg/L Eu/CDs@SiO_2_@IIPs aqueous solutions with pH values ranging from 3.0 to 13.0 were prepared as test solutions. After equilibration until no obvious fluorescence change was observed, the relative fluorescence intensities at different pH values were measured. As shown in Figure 7b, Eu/CDs@SiO_2_@IIPs exhibited significant fluorescence quenching under acidic conditions, while the relative fluorescence intensity was strongest in the range of pH 7.0–8.0, indicating that Eu/CDs@SiO_2_@IIPs are more suitable for operation under near-neutral conditions. Figure 7c shows the relative fluorescence intensity of a 31 mg/L Eu/CDs@SiO_2_@IIPs aqueous solution within 60 min under static conditions. The fluorescence intensity remained nearly unchanged during this period, and repeated tests confirmed that the fluorescence stability could be maintained for at least 60 min, meeting the requirements for detection. To clarify the timescale of detection, the fluorescence behavior of Eu/CDs@SiO_2_@IIPs toward 50 nM Cu^2+^ was examined. As presented in Figure 7d, the fluorescence (31 mg/L) decreased sharply upon Cu^2+^ exposure and reached equilibrium within 3.0 min, demonstrating that the system responds within this timespan.

### 2.4. Fluorescence Detection Performance of Eu/CDs@SiO_2_@IIPs for Cu^2+^

**Figure 8:**
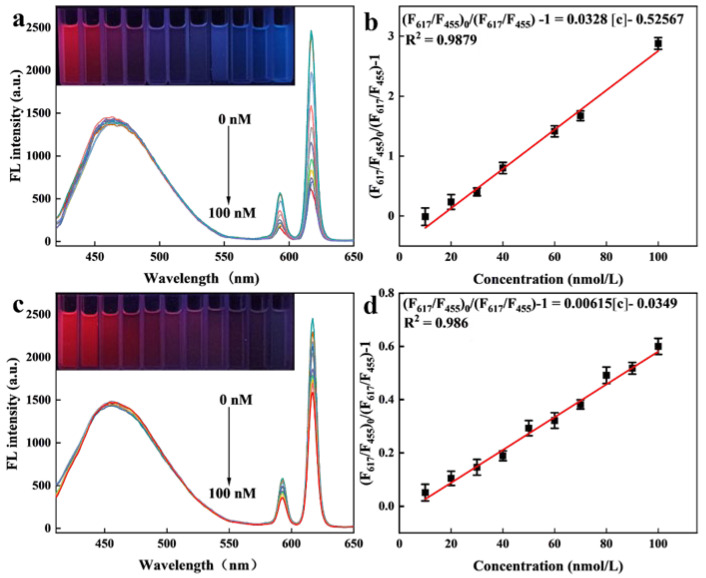
Fluorescence spectra of Eu/CDs@SiO_2_@IIPs (**a**) and Eu/CDs@SiO_2_@NIPs (**c**) in the concentration range of Cu^2+^ from 0 to 100 nM (The inset shows the actual fluorescence color change in the assay solution at the corresponding concentration); Linear relationship of Eu/CDs@SiO_2_@IIPs (**b**) and Eu/CDs@SiO_2_@NIPs (**d**) upon the same concentration range of Cu^2+^.

The primary aim of this work was to assess the detection performance and visual response of Eu/CDs@SiO_2_@IIPs toward Cu^2+^, while the detection process was performed under optimized conditions. Specifically, Cu^2+^ solutions with concentrations ranging from 0 to 100 nM were prepared as test solutions, into which Eu/CDs@SiO_2_@IIPs and Eu/CDs@SiO_2_@NIPs were dispersed at a fixed concentration of 31 mg/L. Fluorescence spectra were recorded at an excitation wavelength of 357 nm. Linear regression analysis was performed to construct calibration curves correlating Cu^2+^ concentration with fluorescence response, and the Stern–Volmer equation was employed to establish the regression model. Figure 8 presents the fluorescence spectra of Eu/CDs@SiO_2_@IIPs (a) and Eu/CDs@SiO_2_@NIPs (c) at different Cu^2+^ concentrations (0–100 nM), together with the corresponding linear relationships. As shown in Figure 8a, with increasing Cu^2+^ concentration, the fluorescence intensity of Eu/CDs@SiO_2_@IIPs at 617 nm decreased markedly, whereas the intensity at 455 nm remained nearly unchanged, accompanied by a visible color change in the detection solutions (insets). The fluorescence quenching efficiency, defined as (F_617_/F_455_)_0_/(F_617_/F_455_) − 1, was plotted against Cu^2+^ concentration to evaluate the linear relationship. As shown in Figure 8b, the calibration equation of Eu/CDs@SiO_2_@IIPs was Log[(F_617_/F_455_)_0_/(F_617_/F_455_) − 1] = 0.0328[c] − 0.5267 with a correlation coefficient (R^2^) of 0.9879. The limit of detection (LOD) and limit of quantification (LOQ) were calculated to be 2.79 nM and 9.37 nM, respectively, defining an effective detection range of 10–100 nM. These results indicate that the fluorescence quenching behavior of Eu/CDs@SiO_2_@IIPs is specifically associated with Cu^2+^, and the sensor exhibits a reliable linear response to Cu^2+^ concentration, demonstrating its potential application in monitoring Cu^2+^ in aquatic environments.

The fluorescence detection performance of Eu/CDs@SiO_2_@NIPs was evaluated under the same conditions as Eu/CDs@SiO_2_@IIPs. Figure 8c shows the fluorescence spectra of Eu/CDs@SiO_2_@NIPs at Cu^2+^ concentrations ranging from 0 to 100 nM, along with the corresponding fluorescence color changes in the detection solutions. The linear relationship is presented in Figure 8d, with the calibration equation Log[(F_617_/F_455_)_0_/(F_617_/F_455_) − 1] = 0.00615[c] − 0.0349 and a correlation coefficient (R^2^) of 0.986. A comparison between Figure 8a and Figure 8c clearly demonstrates that the fluorescence response of Eu/CDs@SiO_2_@IIPs toward Cu^2+^ is much stronger than that of Eu/CDs@SiO_2_@NIPs. This result confirms that the imprinted cavities generated after template removal in Eu/CDs@SiO_2_@IIPs effectively enhance the adsorption and recognition of Cu^2+^. In addition, the distinct fluorescence color changes observed in the insets further indicate that Eu/CDs@SiO_2_@IIPs provide superior visual recognition performance compared with Eu/CDs@SiO_2_@NIPs.

### 2.5. Selectivity Assessment of Eu/CDs@SiO_2_@IIPs

**Figure 9:**
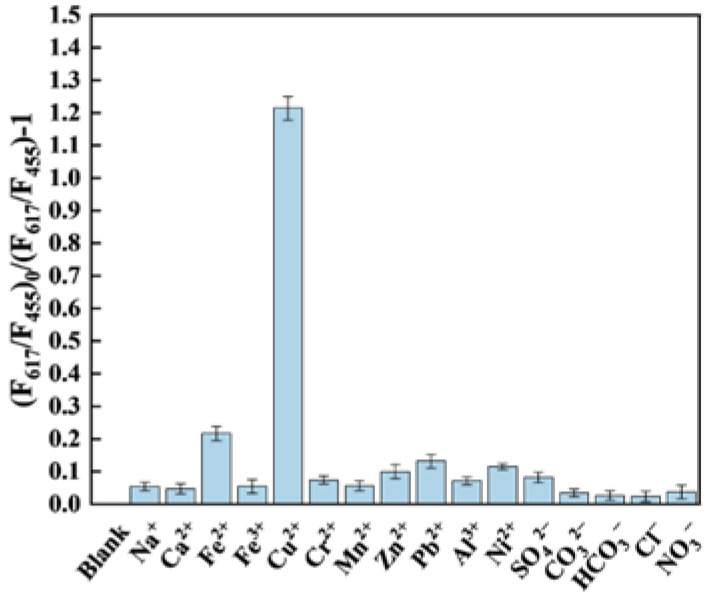
Fluorescence quenching of Eu/CDs@SiO_2_@IIPs in the presence of 50 nM representative inorganic ions.

The selectivity of Eu/CDs@SiO_2_@IIPs is critical for their application in complex environmental samples. The potential interference from background electrolytes was evaluated by considering representative cations and anions typically found in natural waters. As shown in Figure 9, compared with previously reported ion-imprinted fluorescence sensors using APTES as the functional monomer, Eu/CDs@SiO_2_@IIPs exhibited superior selectivity against interfering ions such as Fe^2+^, Zn^2+^, and Pb^2+^. Although Fe^2+^ typically induces chromogenic responses through interaction with phenanthroline rings, the introduction of the imprinted layer substantially strengthened the selective recognition of Cu^2+^, thereby minimizing Fe^2+^ interference under equal concentrations. These results demonstrate that Eu/CDs@SiO_2_@IIPs possess strong selectivity toward Cu^2+^ even in the presence of competing ions, enabling their application in the detection of trace Cu^2+^ in complex real water samples.

### 2.6. Mechanistic Study of Fluorescence Quenching

**Figure 10:**
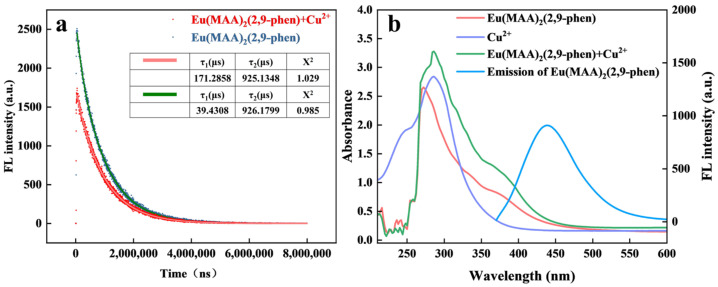
Fluorescence lifetimes of Eu(MAA)_2_(2,9−phen) and Eu(MAA)_2_(2,9−phen) with 50 nM Cu^2+^ (**a**). Fluorescence emission spectrum of Eu(MAA)_2_(2,9−phen), UV absorption spectra of Eu(MAA)_2_(2,9-phen), Cu^2+^ and Eu(MAA)_2_(2,9−phen) with 50 nM Cu^2+^ (**b**).

The fluorescence quenching mechanism of Eu/CDs@SiO_2_@IIPs with Cu^2+^ was explored through fluorescence lifetime, UV–vis absorption, and zeta potential analyses. Figure 10a presents the transient fluorescence spectra of Eu(MAA)_2_(2,9-phen) in the absence and in the presence of 50 nM Cu^2+^. It can be observed that the fluorescence lifetime of Eu(MAA)_2_(2,9-phen) changed significantly upon the introduction of Cu^2+^. The quenching type was further determined by calculating the fluorescence quenching rate constant (Kq) based on the Stern–Volmer relationship.

(1) $$\frac{\tau_{0}}{\tau }-1=K_{q}\tau_{0}\left[C\right]=K_{sv}[C]$$

To investigate the fluorescence quenching mechanism between Eu/CDs@SiO_2_@IIPs and Cu^2+^, analyses were carried out using fluorescence lifetime and UV–vis absorption spectra. In particular, the fluorescence lifetime mentioned here refers to the overall fluorescence lifetime of the Eu(MAA)_2_(2,9-phen) complex measured under excitation with a 375 nm laser using a time-resolved fluorescence spectrometer. Figure 10a shows the transient fluorescence spectra of Eu(MAA)_2_(2,9-phen) in the absence and presence of 50 nM Cu^2+^, where the overall decay of the complex becomes faster and the amplitude-weighted average fluorescence lifetime decreases in the presence of Cu^2+^. τ_1_ represents the fluorescence lifetime of Eu(MAA)_2_(2,9-phen) before the addition of Cu^2+^, while τ_2_ is the lifetime after Cu^2+^ addition; Kq denotes the quenching rate constant, and KSV is the Stern–Volmer constant. The calculated Kq value was 5.13 × 10^8^ L·mol^−1^·s^−1^, which is lower than 2.00 × 10^10 L·mol^−1^·s^−1^, indicating that the quenching process is dynamic in nature. As shown in Figure 10b, the UV–vis absorption spectra of Eu(MAA)_2_(2,9-phen), Cu^2+^, and their mixture demonstrated that the absorption peak at 272 nm red-shifted in the presence of Cu^2+^, which was attributed to the coordination between Cu^2+^ and the nitrogen atom of the phenanthroline ring in 2,9-phen. Furthermore, zeta potential analysis revealed that Eu(MAA)_2_(2,9-phen) had a potential of –16.92 mV at pH 7.0, suggesting that it carried a negative charge and could act as an electron donor to Cu^2+^. This interaction initiated a photoinduced electron transfer (PET) process, which interrupted the ligand-to-metal charge transfer (LMCT) from 2,9-phen to the Eu^3+^ center, thereby disrupting the sensitization pathway. Taken together, these results demonstrate that the main quenching mechanisms of Eu/CDs@SiO_2_@IIPs toward Cu^2+^ involve both dynamic quenching and inhibition of the LMCT process.

### 2.7. Evaluation of Response Efficiency and Sensitivity Threshold

To evaluate the fluorescence sensing performance of Eu/CDs@SiO_2_@IIPs, several previously reported lanthanide-based ratiometric fluorescent sensors for Cu^2+^ detection were selected for comparison. The comparison was based on key parameters including detection range, detection limit, and response time, as summarized in Table 2. The results indicate that Eu/CDs@SiO_2_@IIPs exhibit clear advantages in both detection limit and response time. This superior performance can be attributed to the europium complex Eu(MAA)_2_(2,9-phen) used in this work, which integrates both the recognition function and the fluorescent response into a single monomer. Such an integrated design accelerates analyte access to the imprinted sites, minimizes non-specific fluorescence, and thus enables a lower detection limit and a shorter response time. In addition, the selective recognition ability provided by ion imprinting further enhances their applicability for on-site detection of Cu^2+^.

### 2.8. Real Sample Validation

The ultimate objective of preparing Eu/CDs@SiO_2_@IIPs is their application in real sample detection. In this chapter, deionized water and river water were selected as representative samples, and the detection capability of Eu/CDs@ SiO_2_@IIPs was evaluated using the standard addition recovery method, with results further compared to those obtained by ICP-MS. The deionized water samples were collected from the laboratory, while the river water samples were obtained from the Yudai River within Jiangsu University. As shown in Table 3, the recovery rates of spiked Cu^2+^ using Eu/CDs@ SiO_2_@IIPs were 98.6–107% for deionized water and 102–106% for tap water, with relative standard deviations (RSDs) of 3.7–4.4% and 3.6–5.5%, respectively. In addition, the detected Cu^2+^ concentration in electroplating wastewater using Eu/CDs@ SiO_2_@IIPs was consistent with the results obtained by ICP-MS. These findings demonstrate that Eu/CDs@ SiO_2_@IIPs are capable of accurate and rapid detection of Cu^2+^ in real samples.

## 3. Materials and Methods

### 3.1. Materials

The chemical reagents utilised in this study included europium oxide (Eu_2_O_3_, 99.99%), 1,10-phenanthroline-2,9-dicarboxylic acid (2,9-phen, C_14_H_8_N_2_O_4_, analytical grade), methacrylic acid (MAA, C_4_H_6_O_2_, analytical grade), ethylene glycol dimethacrylate (EGDMA, C_10_H_14_O_4_, analytical grade), azobisisobutyronitrile (AIBN, C_8_H_12_N_4_, analytical grade), and 3-(trimethoxysilyl silicon) propyl methacrylate (KH570, C_10_H_20_O_5_Si, analytical grade), all purchased from Aladdin Biochemical Technology Co., Ltd (Shanghai, China). Double distilled water was prepared and used for the cleaning processes. All other chemicals used were analytical grade and obtained commercially.

### 3.2. Apparatus

FT-IR spectra were acquired on a Nicolet iS50 spectrometer (Thermo Scientific, Waltham, MA, USA). The morphology of the samples was investigated using SEM (JSM-7800F, JEOL, Tokyo, Japan) and TEM (JEM-2010HR, JEOL). Fluorescence emission spectra were recorded on an F-98 spectrofluorometer (Shanghai Leng Guang Technology Co., Ltd., Shanghai, China). Elemental analyses (C, H, N, O) were performed using an Elemental Analyzer (FlashSmart, Thermo Fisher Scientific, Berlin, Germany).

### 3.3. Preparation of the Lanthanide Complex Eu(MAA)_2_(2,9-phen)

The polymerizable lanthanide complex Eu(MAA)_2_(2,9-phen) was synthesized as follows. First, 2.2 g of Eu_2_O_3_ was dissolved in 50 mL of dilute hydrochloric acid (6 mol/L) to obtain a 0.25 mol/L EuCl_3_ solution. The product EuCl_3_·was collected, washed three times with anhydrous ethanol, and dried for later use. Subsequently, 0.223 g of EuCl_3_·6H_2_O was dissolved in 50 mL of anhydrous ethanol, followed by the dropwise addition of 1.31 mL of MAA. The pH of the solution was adjusted to 8.0 using NaOH solution, and the mixture was stirred magnetically for 30 min. Then, 0.134 g of 2,9-phen was added to the solution, and the mixture was stirred for 12 h until it turned slightly reddish and exhibited strong red fluorescence under UV light at 365 nm. The product was collected by centrifugation, washed three times with distilled water and anhydrous ethanol, and finally vacuum-dried for further use.

### 3.4. Preparation of CDs

The synthesis of amino-functionalized CDs was carried out according to a previously reported method with slight modifications. Briefly, 0.5 g of anhydrous citric acid and 0.5 g of urea were ultrasonically dissolved in 12.5 mL of deionized water and magnetically stirred for 30 min. The resulting solution was transferred into a 30 mL Teflon-lined stainless-steel autoclave and heated at 160 °C for 5.0 h using a hydrothermal method. Following natural cooling to room temperature, the CD-containing mixture was purified by dialysis against deionized water using a 1000 Da cutoff membrane for three days, with water refreshed at 8 h intervals under magnetic stirring. The dialyzed product was subsequently frozen at −80 °C, freeze-dried, and stored in darkness.

### 3.5. Preparation and Surface Functionalization of CDs@SiO_2_ Nanoparticles

CDs@SiO_2_ nanoparticles were prepared using a reverse microemulsion method. Briefly, 7.5 mL of cyclohexane, 1.8 mL of n-hexanol, and 1.77 mL of Triton X-100 were mixed in a 50 mL three-neck flask, followed by the addition of 3.0 mg of CDs and stirring for 20 min. Then, 0.48 mL of deionized water and 2.0 mL of ammonia solution were introduced, and a turbid microemulsion was formed. Subsequently, 1.67 mL of TEOS was added dropwise, and the reaction was maintained at room temperature under dark and sealed conditions for 24 h. The products were collected by centrifugation and washed with deionized water and ethanol. For surface functionalization, 200 mg of CDs@SiO_2_ was ultrasonically dispersed in 50 mL of anhydrous ethanol, followed by the addition of 2 mL of KH570. After stirring at room temperature for 24 h, the products were separated by centrifugation, washed three times with deionized water and ethanol, and vacuum-dried for further use.

### 3.6. Preparation of Eu/CDs@SiO_2_@IIPs

Eu/CDs@SiO_2_@IIPs were prepared as follows. First, 0.593 g of Eu(MAA)_2_(2,9-phen) and 0.1345 g of CuCl_2_ were ultrasonically dispersed in 20 mL of acetonitrile in a three-neck flask and left to stand for 2 h to form the pre-polymerization system. Separately, 100 mg of vinyl-functionalized CDs@SiO_2_ was ultrasonically dispersed in 30 mL of acetonitrile for 30 min. The two solutions were then combined, followed by the addition of 0.792 mL of the cross-linker EGDMA. After purging with nitrogen for 30 min to remove dissolved oxygen, 10 mg of the initiator AIBN was introduced, and the reaction was carried out under sealed conditions at 65 °C with magnetic stirring for 24 h. The products were collected by centrifugation and subjected to Soxhlet extraction with 0.2 mol/L ammonia solution to remove the template ions (Cu^2+^), followed by washing three times with deionized water and ethanol, and vacuum drying to obtain Eu/CDs@SiO_2_@IIPs. The corresponding non-imprinted polymers (Eu/CDs@SiO_2_@NIPs) were prepared using the same procedure without the addition of Cu^2+^ in the pre-polymerization system.

### 3.7. Fluorescent Detection of Cu^2+^

A stock aqueous solution of Eu/CDs@SiO_2_@IIPs was prepared by ultrasonic dispersion at a concentration of 0.5 g/L and stored in colorimetric tubes. Cu^2+^ solutions with a series of concentrations (10 mL each) were prepared as target solutions. Then, 200 μL of the stock solution was added into each target solution, and fluorescence measurements were carried out using a fluorescence spectrophotometer. The linear relationship between fluorescence intensity of Eu/CDs@SiO_2_@IIPs and Cu^2+^ concentration was established. All fluorescence measurements were performed at room temperature, and the excitation wavelength was set according to the optimal excitation of Eu(MAA)_2_(2,9-phen).

## 4. Conclusions

In this study, a surface molecularly imprinted ratiometric fluorescent sensor (Eu/CDs@SiO_2_@IIPs) was successfully constructed by embedding green and non-toxic carbon dots as internal reference signals and employing a newly synthesized europium complex Eu(MAA)_2_(2,9-phen) as both the fluorescent and functional monomer. The pre-polymerization strategy enabled the functional monomers to be precisely distributed at the imprinted sites, eliminating background fluorescence interference and ensuring that signal variation was solely attributed to Cu^2+^ recognition. As a result, the sensor achieved a rapid response time of 3.0 min, an ultralow detection limit of 2.79 nM, and excellent selectivity against competing ions, owing to the coordination between uncoordinated nitrogen atoms in the phenanthroline ligand and Cu^2+^. The ratiometric dual-emission behavior further allowed reliable visual detection, and validation with real water samples confirmed strong agreement with ICP-MS results. This work demonstrates the effectiveness of incorporating customized rare-earth fluorescent monomers into ion-imprinted sensors, offering a practical platform for the on-site visual detection of trace heavy metal ions in complex environments. In addition, the proposed sensor can be fabricated from inexpensive, commonly available chemical reagents and only a small amount of light rare-earth europium, resulting in a low-cost yet highly effective tool for rapid on-site emergency detection of Cu^2+^ ions.

## Figures and Tables

**Table 1:** Elemental analysis of Eu(MAA)_2_(2,9-phen).

Sample		C (%)	H (%)	N (%)	O (%)
Eu(MAA)_2_(2,9-phen)	found calc.	44.20 44.92	3.65 2.74	5.61 4.76	21.10 21.74

**Table 2:** Comparison of Eu/CDs@SiO_2_@IIPs with other lanthanide ratio fluorescence sensors.

	Sensor Name	Linear Range (nM)	Detection Limit (nM)	Response Time (min)	Bibliography
1	Eu/CDs@SiO_2_@IIPs	10–100	2.79	3.0	this work
2	Ln(dpa)_3_@POSS-NH_2_	1 × 10^4^–1 × 10^7^	not given	30	[41]
3	Eu:AMC(Tb:cs124)-DTPA-PEG-Fe_3_O_4_-Au	30–100	30	10	[42]
4	Glu/Gly/EuCl_3_·6H_2_O	0–50,000	not given	20	[43]
5	Eu(DPA)_3_@UIO-66	0–25,000	890	30	[44]
6	Eu-doped PS@PSS@GSH-CdTe	0–1000	1.45	10	[45]
7	DPA-Eu^3+^-PEI probe	20–10,000	8.0	1.0	[46]

**Table 3:** Evaluation of Cu^2+^ Levels in Drinking Water by Fluorescent Sensor and ICP-MS.

Eu/CDs@SiO_2_@IIPs							ICP-MS	
	Sample Number	Add Concentration (nM)	Detection Limit (nM)	Recovery Rate (%)	RSD (n = 3)	Detection Limit (μM)	Recovery Rate (%)	RSD (n = 3)
Deionized water	1			103.7	3.2	20.2	101	1.3
	2	20	20.74	96.9	4.4	51	102	2.1
	3	50	48.48	102.1	3.2	102	102	1.1
Creek	1	100	102.1	104	1.2	20.4	102	1.6
	2	20	20.8	107	4.5	50.5	102	1.2
	3	50	53.5	99.3	2.4	103	101	2.5
Electroplating wastewater	1	100	115.2	-	2.8	112.5	103	2.2
	2	0	127.6	-	4.3	117.4	-	1.7
	3	0	109.3	-	3.7	113.9	-	1.3

## Data Availability

The original contributions presented in this study are included in the article. Further inquiries can be directed to the corresponding author(s).

## References

[1] Rehman M., Liu L., Wang Q., Saleem M.H., Bashir S., Ullah S., Peng D. (2019). Copper environmental toxicology, recent advances, and future outlook: A review. Environ. Sci. Pollut. Res..

[2] Zeb M., Khan K., Younas M., Farooqi A., Cao X., Kavil Y.N., Alelyani S.S., Alkasbi M.M., Al-Sehemi A.G. (2024). A review of heavy metals pollution in riverine sediment from various Asian and European countries: Distribution, sources, and environmental risk. Mar. Pollut. Bull..

[3] Shi J., Zhao D., Ren F., Huang L. (2023). Spatiotemporal variation of soil heavy metals in China: The pollution status and risk assessment. Sci. Total. Environ..

[4] Bandara G.C., Heist C.A., Remcho V.T. (2018). Chromatographic separation and visual detection on wicking microfluidic devices: Quantitation of Cu^2+^ in surface, ground, and drinking water. Anal. Chem..

[5] Lu Y., Wei X., Chen M., Wang J. (2023). Non-ceruloplasmin-bound copper and copper speciation in serum with extraction using functionalized dendritic silica spheres followed by ICP-MS detection. Anal. Chim. Acta.

[6] Bahçıvan A., Şaylan M., Sagdic O., Bakırdere S. (2024). CoSn(OH)_6_ nanocubes as a solid sorbent for the effective preconcentration of copper ions in cinnamon (*Cinnamomum zeylanicum*) extract. Food Chem..

[7] Zhou F., Wu B., Zhou J. (2024). Novel Spectrophotometric Method for Robust Detection of Trace Copper and Cobalt in High-Concentration Zinc Solution. Molecules.

[8] Bettini S., Pagano R., Valli L., Giancane G. (2014). Spectroscopic investigation of the selective interaction of mercuric and cupric ions with a porphyrin active layer. J. Phys. Chem. C.

[9] Wei J., Li J., Jiang Z., Tang Z., Guo P., Zhao Y.-L., Ke P., Wang A. (2023). Improved electrochemical detection of copper ions in nitrogen doped ta-C films for marine corrosive monitoring. Electrochim. Acta.

[10] Liu S., Wang Y.-M., Han J. (2017). Fluorescent chemosensors for copper(II) ion: Structure, mechanism and application. J. Photochem. Photobiol. C Photochem. Rev..

[11] Chen J., Li Z., Li X., Du Y. (2024). A reverse-signal response fluorescence sensor based on NH2-MIL-101(Fe) and silicon-doped carbon quantum dots for selective determination of copper ions. J. Photochem. Photobiol. A Chem..

[12] Li J., Zhao L., Zhang T., Yang Z., Liu X., Hu B., Zhao L., Che G. (2025). Fabrication of Mesoporous ion-Imprinted Fluorescent Sensors for Rapid and Reliable Cu^2+^ Detection in Aqueous Environments. Results Surf. Interfaces.

[13] Hassan M., Zareef M., Xu Y., Li H., Chen Q. (2021). SERS based sensor for mycotoxins detection: Challenges and improvements. Food Chem..

[14] Li J., Li C., Guo W., Guo Y., Zou X., Sun Z. (2025). Recyclable magnetic HNTs@MIPs-Based SERS sensors for selective, sensitive, and reliable detection of capsaicin for gutter oil discrimination. Food Biosci..

[15] Feng N., Dong C., Shuang S., Song S. (2025). Fluorescence and colorimetric dual-mode sensing of copper ions and fingerprint visualization by benzimidazole derivatives. Spectrochim. Acta Part A Mol. Biomol. Spectrosc..

[16] Hu B., Zhao W., Chen L., Liu Y., Ma Z., Yan Y., Meng M. (2024). Enhanced molecularly imprinted fluorescent test strip for rapid and visual detection of norfloxacin via a smartphone. Molecules.

[17] Lin K.-T., Nian X., Li K., Han J., Zheng N., Lu X., Guo C., Lin H., Jia B. (2023). Highly efficient flexible structured metasurface by roll-to-roll printing for diurnal radiative cooling. eLight.

[18] Minopoli A., Wagner S., Erben E., Liao W., Stoev I.D., Lauga E., Kreysing M. (2023). ISO-FLUCS: Symmetrization of optofluidic manipulations in quasi-isothermal micro-environments. eLight.

[19] Zhang C., Shi X., Yu F., Quan Y. (2020). Preparation of dummy molecularly imprinted polymers based on dextran-modified magnetic nanoparticles Fe3O4 for the selective detection of acrylamide in potato chips. Food Chem..

[20] Bai Q., Huang C., Ma S., Gong B., Ou J. (2023). Rapid adsorption and detection of copper ions in water by dual-functional ion-imprinted polymers doping with carbon dots. Sep. Purif. Technol..

[21] Dong H., Ran C., Gao W., Li M., Xia Y., Huang W. (2023). Metal Halide Perovskite for next-generation optoelectronics: Progresses and prospects. eLight.

[22] Bai Y., Lu H., Lei M., Qiu J., Lin J. (2025). An ultrastable luminescent covalent organic polymer for selective Pd^2+^ detection in strong acid. EcoEnergy.

[23] Ahmad W., Aljuhani E., Alwael H., Assirey E., Nassef H., El-Shahawi M. (2023). Redox impulse, computational calculation of molecular energy potentials and ultra-trace determination of the food colorant erythrosine b in fruit jams, soft drinks and water. J. Food Compos. Anal..

[24] Xu Y., Huang T., Wang S., Yan Y. (2022). Mesoporous silica-based molecularly imprinted fluorescence sensor for the ultrafast and sensitive recognition of oxytetracycline. J. Food Compos. Anal..

[25] Hu B., Chen L., Yu Z., Xu Y., Dai J., Yan Y., Ma Z. (2021). Hollow molecularly imprinted fluorescent sensor using europium complex as functional monomer for the detection of trace 2,4,6-trichlorophenol in real water samples. Spectrochim. Acta Part A Mol. Biomol. Spectrosc..

[26] Wang Y., Xu W., Liu H., Jing Y., Zhou D., Ji Y., Widengren J., Bai X., Song H. (2024). A multiband NIR upconversion core-shell design for enhanced light harvesting of silicon solar cells. Light. Sci. Appl..

[27] Nguyen M.-C., Nguyen H.-Q., Kang H., Goddati M., Lee S.-Y., Yee K.-J., Lee J. (2023). Metal plasmon-enhanced lanthanide fluorescent nanoparticles for monitoring aqueous copper ions. Mater. Today Nano.

[28] Yang L., Hu W., Pei F., Liu Z., Wang J., Tong Z., Mu X., Du B., Xia M., Wang F. (2024). A ratiometric fluorescence imprinted sensor based on N-CDs and metal–organic frameworks for visual smart detection of malathion. Food Chem..

[29] Fan Y., Huang W., Zhu F., Liu X., Jin C., Guo C., An Y., Kivshar Y., Qiu C.-W., Li W. (2024). Dispersion-assisted high-dimensional photodetector. Nature.

[30] Chen X., Shu X., Zhou J., Wan L., Xiao P., Fu Y., Ye J., Huang Y.-T., Yan B., Xue D. (2024). Additive engineering for Sb2S3 indoor photovoltaics with efficiency exceeding 17%. Light. Sci. Appl..

[31] Bettini S., Syrgiannis Z., Pagano R., D̵oRd̵eVić L., Salvatore L., Prato M., Giancane G., Valli L. (2019). Perylene bisimide aggregates as probes for subnanomolar discrimination of aromatic biogenic amines. ACS Appl. Mater. Interfaces.

[32] Han E., Pan Y., Li L., Cai J. (2023). Bisphenol A detection based on nano gold-doped molecular imprinting electrochemical sensor with enhanced sensitivity. Food Chem..

[33] Dong Z., Lu J., Wu Y., Meng M., Yu C., Sun C., Chen M., Da Z., Yan Y. (2020). Antifouling molecularly imprinted membranes for pretreatment of milk samples: Selective separation and detection of lincomycin. Food Chem..

[34] Su X., Zheng K., Tian X., Zhou X., Zou X., Xu X., Sun Z., Zhang W. (2023). An advanced ratiometric molecularly imprinted sensor based on metal ion reoxidation for indirect and ultrasensitive glyphosate detection in fruit. Food Chem..

[35] Zhou T., Ding L., Che G., Jiang W., Sang L. (2019). Recent advances and trends of molecularly imprinted polymers for specific recognition in aqueous matrix: Preparation and application in sample pretreatment. TrAC Trends Anal. Chem..

[36] Yang C., Hu W., Liu J., Han C., Gao Q., Mei A., Zhou Y., Guo F., Han H. (2024). Achievements, challenges, and future prospects for industrialization of perovskite solar cells. Light. Sci. Appl..

[37] Xie F., Jin W., Nolen J.R., Pan H., Yi N., An Y., Zhang Z., Kong X., Zhu F., Jiang K. (2024). Subambient daytime radiative cooling of vertical surfaces. Science.

[38] Cai Y., Cao L., Cai H., Yang W., Lu H., Adila A., Zhang B., Cao Y., Huang W., Xu W. (2025). A rapid microfluidic paper-based chip sensor using ratiometric fluorescence and molecularly imprinted polymers for visual detection of sulfadiazine in actual samples. J. Food Compos. Anal..

[39] Qiu H., Gao L., Wang J., Pan J., Yan Y., Zhang X. (2017). A precise and efficient detection of Beta-Cyfluthrin via fluorescent molecularly imprinted polymers with ally fluorescein as functional monomer in agricultural products. Food Chem..

[40] Zhou T., Wang Y., Li T., Li H., Yang C., Sun D., Wang D., Liu C., Che G. (2021). Fabricating magnetic hydrophilic molecularly imprinted resin with enhanced adsorption and recognition performance for targeted detecting chlorophenols in environmental water. Chem. Eng. J..

[41] Xu Q., Li Z., Li H. (2016). Water-Soluble Luminescent Hybrid Composites Consisting of Oligosilsesquioxanes and Lanthanide Complexes and their Sensing Ability for Cu^2+^. Chem.—A Eur. J..

[42] Liu J., Liu J., Liu W., Zhang H., Yang Z., Wang B., Chen F., Chen H. (2015). Triple-emitting dumbbell fluorescent nanoprobe for multicolor detection and imaging applications. Inorg. Chem..

[43] Ren H., Wang X., Gong R., Li M., Zhu H., Zhang J., Duan E. (2020). Atomically dispersed Eu(III) sites in natural deep eutectic solvents based fluorescent probe efficient identification of Fe^3+^ and Cu^2+^ in wastewater. Spectrochim. Acta Part A Mol. Biomol. Spectrosc..

[44] Chen Z., Zhang W., Wen X. (2024). A dual-emission ratiometric luminescence probe based on Zr-MOFs for the efficient detection of Cu^2+^ in aqueous solutions. Funct. Mater. Lett..

[45] Ye Z., Li L., Zhao F., Yang Q., Wang Y., Bohinc K., Guo X. (2020). Dual-emission fluorescent probe templated by spherical polyelectrolyte brush for ratiometric detection of copper ions. J. Mater. Sci..

[46] Wang J., Pei J., Li G. (2023). Lanthanide ternary complex as a fluorescent probe for highly sensitive and selective detection of copper ions based on selective recognition and photoinduced electron transfer. Spectrochim. Acta Part A Mol. Biomol. Spectrosc..

